# The Use of Diaphragm Ultrasonography in Pulmonary Physiotherapy of COPD Patients: A Literature Review

**DOI:** 10.3390/jcm9113525

**Published:** 2020-10-31

**Authors:** Agnieszka Lewińska, Karen Shahnazaryan

**Affiliations:** 1 Faculty of Rehabilitation, Józef Piłsudski University of Physical Education in Warsaw, 00-968 Warsaw, Poland; 2 Department of Rehabilitation, Medical Faculty, Medical University of Warsaw, 02-091 Warsaw, Poland; karen.shahnazaryan@wum.edu.pl

**Keywords:** diaphragm, physiotherapy, rehabilitation, COPD, ultrasound

## Abstract

There is potentially a broad range of patient populations in which ultrasound imaging (US) might be beneficial form of physiotherapy process support. Among them, the group of patients with chronic obstructive pulmonary disease (COPD) is of great importance, as in this individuals the diaphragm dysfunction is frequently observed. Pulmonary physiotherapy often includes techniques which are intended to influence the diaphragm muscle but its anatomy does not allow for variety of techniques to assess function. Lack of easily available and reliable measures complicates outcomes interpretation and makes decision-making process difficult. A review of the electronic literature was conducted to identify studies related to the US assessment of physiotherapy process and its outcome in COPD patients. As a consequence, seven papers were identified. Based on the results obtained, it can be concluded that the diaphragm excursion is US measure that is most often described in context of diaphragm-related physiotherapy in COPD patients. The methodology applied, however, varies greatly making it difficult to compare results. Thus, developing standards of outcome assessment methods and therapy monitoring systems which are supported by evidence should be of paramount importance. Future studies could also focus on identifying which components of physiotherapeutic diaphragm-targeted approach provide acceptable level of evidence.

## 1. Introduction

Wide range of patient populations potentially exists in which ultrasonography (US) might be beneficial form of physiotherapy process support. Among them, the group of patients suffering from chronic obstructive pulmonary disease (COPD) is of great importance, as its high prevalence, morbidity, and mortality is a growing challenge for health-care systems. There was relationship demonstrated between altered mobility of the diaphragm and parameters that quantify air trapping (residual volume and residual volume to total lung capacity ratio) in people with COPD [1]. The decline in diaphragmatic motion reflecting impaired respiratory muscle function is a significant risk factor for increased mortality. A mechanical strain which is imposed on the inspiratory muscles, is caused by increased resistive and elastic loads, resulting from greater airway resistance and reduced dynamic pulmonary compliance. Thoracic hyperinflation caused by air trapping changes diaphragm muscle fibers orientation in a zone of apposition (ZOA), which makes the contraction less effective at lower rib cage expansion. There is also a reduction in the number of sarcomeres to restore its pressure-generating capacity. The remodeling results in flattening of the muscle and subsequent decreased diaphragmatic excursion [2].

Still, dysfunction of the diaphragm appears to be an undervalued cause of respiratory difficulties in COPD patients and it is not commonly reported in the literature regarding physiotherapeutic interventions. Irrespective of that, physiotherapy which serves as an important and beneficial component of PR management, very often includes techniques that directly or indirectly involve the diaphragm muscle [3,4,5,6,7,8,9,10]. The rationale for implementing physiotherapy in COPD patients with diaphragmatic dysfunction is associated with improvement of the quality and effectiveness of ventilation, contributing to exercise capacity increase. That reduces the risk of general functional deterioration.

Each implemented intervention requires the use of assessment and monitoring methods. They need to be accurate, reliable, and reproducible, but also supported by evidence. Diaphragm anatomy however does not allow for a wide range of techniques to assess its structure or function. A several static and dynamic techniques are available [11,12], but it appears that the most accurate and convenient diaphragm assessment method is ultrasound imaging that provides a noninvasive real-time functional visualization, even at a bedside. It allows for assessment of diaphragmatic displacement and thickness, and more importantly inspiratory thickening, which reflects diaphragm contractile activity (during diaphragm contraction the muscle thickens, and this thickening quantifies as the thickening fraction). The technique can also be used to detect structural changes, such as atrophy or load-induced injuries. Most importantly, the procedure is well defined and validated. The normal values and cutoff points for diaphragmatic excursion during normal respiration, forced respiration, and voluntary sniffing have been numerously described for gender and different age groups, as well as diaphragm thickness and thickening ratio values [13].

**Figure 1:**
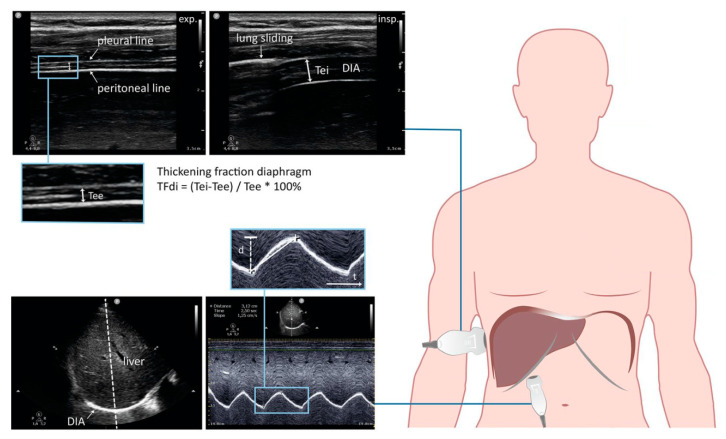
Clinical application of diaphragm muscle ultrasound (Figure modified according to: Tuinman et al. [14], Figure 1; p. 596).

It can be stated that sonographic evaluation can demonstrate the muscle function with quite simple visual image. Diaphragm is usually visualized by ultrasound via two approaches. The right mid-axillary intercostal approach at the zone of apposition, and the subcostal approach using the liver as an acoustic window are summarized in Figure 1.

The images are obtained in brightness mode (B-mode) and motion mode (M-mode). The excursion of hemi-diaphragms is usually measured subcostal using M-mode US, which is a reproducible method in standing and supine position. The liver is used as a window when recording motion of right hemi-diaphragm and the spleen window is used to obtain image of the left one (spleen is however considered poor acoustic window in general). The inspiratory and expiratory displacements of diaphragm (seen as a bright line), lead to shortening and lengthening of the probe-diaphragm distance. A thickness of hemi-diaphragms and inspiratory thickening can be measured directly from the frozen B-mode images. Both hemi-diaphragms can be visualized in the ZOA. Diaphragm is identified then as a three-layered structure. (Two parallel echogenic lines, the diaphragmatic pleura and the peritoneal fascia, enclose the hypoechoic diaphragmatic muscle, and a third hyperechoic line, the fibrous layer in the center of the diaphragm, is seen in the middle of non-echogenic layer) [14,15].

The review was focused on providing an insight into US imaging of diaphragm function in physiotherapeutic treatment and assessment of COPD patients. The primary objective of the review conducted was to identify existing studies, and review the ultrasonography-related methodology applied. That seems important in terms of values obtained interpretation, outcome achieved comparison, and future standardized protocol development. The main question that paper aims to provide answer for, regards comparability of the results. As the US may potentially allow to point the most adequate technique to use in particular patient in given situation, and provide significantly higher accuracies than conventional physiotherapeutic evaluation the secondary aim of the review was to find out whether ultrasound can detect changes in diaphragm function in the course of physiotherapy in COPD patients.

## 2. Materials and Methods

The review was conducted in accordance with the PRISMA statement. An electronic search of the Medline, EMBASE, PEDro, and Cochrane Central Register of Controlled Trials (CENTRAL) databases from inception until September, 2020 was conducted. The literature search was extended by manual searching of reference lists of the essential articles. All references were exported with the use of EndNote X9 and after eliminating duplicates, the identified study titles were screened. For the adequate ones the abstracts were obtained, followed subsequently by full-text examining for relevant content. The search was conducted by two independent reviewers and limited to papers in English language. The first author extracted the information regarding author names, year of publication, intervention received, and US measurements conducted from each article.

Selection criteria were set on primary literature. Studies were included when apply to COPD patients, involve diaphragm ultrasound imaging (as initial evaluation, outcome assessment or feedback, or support of therapy), and concerns physiotherapeutic approaches affecting the diaphragm muscle. Exclusion criteria involved language limitations. Research abstracts from conference and review articles were not included. Two reviewers assessed inclusion and exclusion criteria for each article independently. Any disagreements were resolved on a base of consensus. The following MeSH terms and keywords were used in the search: diaphragm, physiotherapy, rehabilitation, ultrasonography, COPD, respiratory muscle training, breathing exercise, manual therapy, kinesiology taping. The search strategy used for each database is detailed in Appendix A.

All included clinical trials were independently assessed for the risk of bias. Revised Cochrane risk-of-bias tool for randomized trials (RoB2) [16] and Risk Of Bias In Non-randomized Studies of Interventions (ROBINS-I) were used for the assessment [17]. Any disagreements were resolved by consensus. Risk-of-bias VISualization (robvis) [18] was used to create risk-of-bias plots.

**Figure 2:**
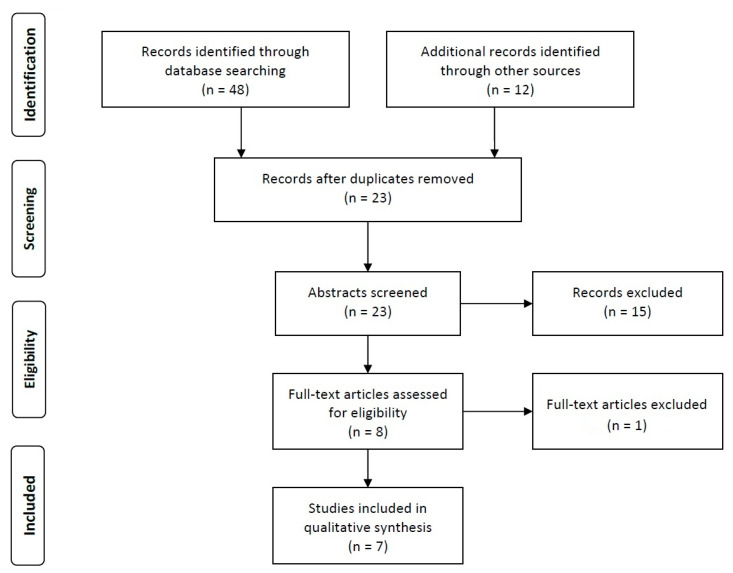
Flow chart of the literature search.

The searching and selection process are demonstrated in the flow chart in Figure 2.

## 3. Results

The literature search identified 48 potentially related articles. References of selected articles were evaluated then, and another 12 potential articles were identified. After excluding duplications (n = 37) 23 articles remained for screening against inclusion criteria. Two papers did not meet the language criteria. Eleven studies were excluded because they did not concern physiotherapeutic intervention, one study did not describe the use of diaphragm US, and four studies turned out to be review articles. Finally, seven studies were included in the qualitative review.

**Figure 3:**
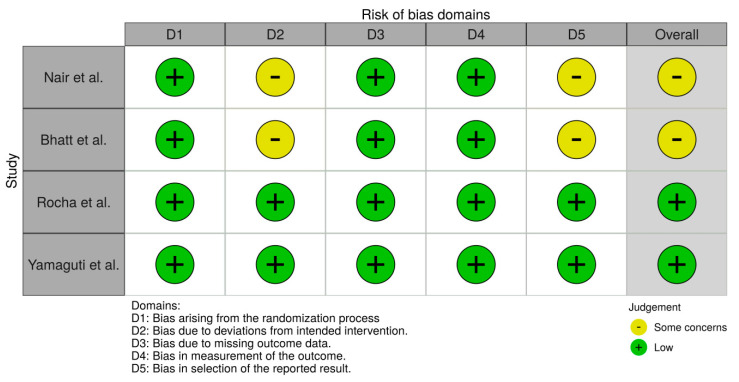
Risk of bias plot according to RoB2.

**Figure 4:**
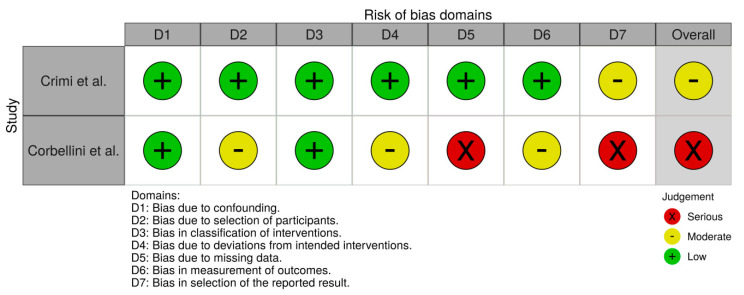
Risk of bias plot according to ROBINS-I.

According to RoB2, studies by Nair et al. and Bhatt et al. had some concerns and studies by Rocha et al. and Yamaguti et al. had a low risk of bias. No trial was able to blind therapists. According to ROBINS-I prospective observational studies assessment, Crimi et al. and Corbellini et al. studies was considered as moderate and serious risk of bias, respectively. A summary of the risk of bias assessment for each study is presented in Figure 3 and Figure 4.

Table 1 summarizes the papers reviewed. The information regarding authors, study design, type and duration of intervention, number of participants, techniques used, outcome measure, and outcome measure details were provided.

Crimi et al. [21] suggested that US is a complementary tool that could be used by physiotherapists in daily clinical practice as the measurements are accurate and reproducible. The technique of assessment is easy to learn. They conducted diaphragm excursion and ZOA parameters, as a form of endurance and resistance training program evaluation. Their primary finding was an increase in diaphragm displacement observed after completing the rehabilitation program. They also found that change in the length of ZOA has a potential to predict positive outcome of exercise training program.

In another study which focused on comprehensive PR impact, Corbelini et al. [22] assessed diaphragmatic excursion in similar circumstances. They demonstrated that patients who completed rehabilitation program showed statistical and clinical increase in diaphragmatic mobility during forced respiration. As this improvement correlated with the increase in inspiratory capacity, it was possible then to determine patients’ dynamic hyperinflation reverse. Similarly to previously described study, they obtained images at right hemi-diaphragm, but the measures were taken when patients were in a semi-recumbent position while Crimi et al. [21] positioned their patients in sitting.

There are US measures of diaphragmatic kinetics that were proposed to define COPD progression [26] and prognosis of PR outcomes [21], but still relatively little is known about the PR effect on diaphragm excursion, thickness, cross sectional area, and echo intensity. A promising outcome can be expected from the future longitudinal study planned by Marques et al. [19]. They intend to analyze short-term effects of PR on the structure and motion of diaphragm, as well as changes over time. They did not provide detailed insight on US protocols planned to be used, but the range of measures is impressive and it may be assumed that the study will provide very important insight on diaphragm functional parameters. It will however be difficult to link the changes observed to a particular intervention, as the study concerns a textbook multidisciplinary PR program.

Yamagutti et al. [25] though, focused on single technique of Diaphragmatic Breathing. They used B-mode examination to assess left branch of the portal vein displacement [27] as a measure of diaphragm excursion during forced respiration. They demonstrated increase in diaphragm mobility in a group of patients performing Diaphragmatic Breathing technique. They also connected mobility improvement to noticeable decrease in dyspnea. Moreover, they stated that breathing exercise conducted, led to advance in exercise tolerance, which is consistent with their previous report on lower exercise tolerance in patients with reduced diaphragmatic mobility [28]. In this case patients were evaluated in the supine position.

Bhatt et al. [24] perform their measurements while patients stay in horizontal position likewise. They also assessed diaphragmatic movements in B-mode ultrasound, but with the use of different approach, that allows obtaining a longitudinal plane of the right hemi-diaphragm [29]. They focused on Pursed Lips Breathing technique and assessed short-term effect of the exercise by measuring diaphragm excursion during normal and forced respiration. Their observation suggested that the worse functional capacity patients presented, the larger increase in diaphragmatic excursion they showed following Pursed Lips Breathing. It was also noticed that diaphragm mobility measured at Forced Vital Capacity correlates with exercise capacity suggesting the potential role of Pursed Lips Breathing in respiratory distress management.

In addition to physical training and breathing exercise, the effect of manual therapy on diaphragm mobility was assessed as well. Nair et al. [20] used B-mode imaging [30] to evaluate immediate effect of Diaphragmatic Stretch and Manual Diaphragm Release techniques. Diaphragm displacement was recorded bilaterally, during forced respiration, in patients staying in sitting position. There was a significant difference found in the range of diaphragmatic excursion following Diaphragmatic Stretch and Manual Diaphragm Release, however without any distinctive changes between these two techniques. Increase in chest expansion was also observed.

Similar outcome of increase in diaphragm mobility after Manual Diaphragm Release was stated by Rocha et al. [23]. They chose different approach though, using M-mode [31] and positioning patients in semi-recumbent. The measures were also obtained during forced respiration.

## 4. Discussion

In the context of papers identified, it appears that diaphragm excursion was the measure most commonly used to assess diaphragm function in patients with COPD. No study on the use of ultrasound as feedback or determinant of most accurate treatment method was identified. Diaphragm ultrasound was used only as a tool to evaluate intervention effectiveness or over-time changes. Physiotherapeutic interventions conducted in the studies included either physical training or breathing exercise, as well as manual therapy. In addition, comprehensive multidisciplinary PR programs were described. In a presence of such diversity of interventions and with concurrent low number of papers searched, it would be hard to present systematized conclusions regarding effectiveness and compare results.

However, diaphragm US performed in COPD patients proved to be relevant technique, able to quantitate their functional impairment and detect improvement in the course of physiotherapy. The studies searched highlight the importance of diaphragm muscle evaluation. The evaluation protocols however differ regarding patients positioning, modes used, approach, and hemi-diaphragm chosen. The lack of standardized procedure not only affects the possibility of drawing broader conclusions, but also preclude routine use of diaphragm US. Diaphragm contractility depends on respiratory demands, as well as on demands of maintaining the body position [32], thus the comparison of outcomes is not reliable when measures compared are taken under different conditions. Similarly is inference about diaphragm thickness on the basis of comparing parameters between different subjects’ right and left hemi-diaphragm, due to side-to side variations [33]. The data obtained from the images of different mode and approach are also incomparable.

As every identified study used diaphragm US as a form of outcome assessment method, it seems that the attention should be paid to the possibility of using US in the course of therapy. In COPD patients abnormal diaphragm activity leads to respiratory difficulties, increasing the role of accessory muscles. Their excessive use, in turn, causes elevated oxygen demand that is difficult to meet as the disease progresses [34]. Early recognition and implementation of the treatment that focuses at diaphragm strength and alignment, may help to reduce the work of respiratory accessory muscles. Determining diaphragmatic movement can also improve the effectiveness of exercise training, while knowing that decreased mobility of diaphragm is associated with reduced exercise tolerance and increased dyspnea upon exertion in COPD patients. Furthermore, as reduced diaphragmatic mobility in COPD patients is related to the air volume possible to exhale [28], defining the extension of this mobility limitation may be useful while choosing breathing exercise and strategies targeted at reduction of air trapping [35]. Considering that combination of diaphragm excursion measures could be used as the predictor of extubation outcome in mechanically ventilated patients with COPD [36], and that reduced diaphragm thickness is associated with reduced chances of weaning from mechanical ventilation [37], necessity of implementing treatment strategies aiming at improving diaphragm function seems evident. Such strategies come down to targeted and individualized respiratory muscle training that requires properly adjusted and systematically modified parameters [38].

Although physiotherapeutic thoracic ultrasound imaging is not a new concept, physiotherapists do not routinely use diaphragm US. Its application in physiotherapy was previously described [39], but there are no standardized recommendations for education in this field. Ultrasound imaging is operator dependent and in general requires significant expertise. Little information exists on the extent of training required for independent practice of non-physician healthcare professionals, but it was suggested that they can be successfully trained in the area of thoracic ultrasound imaging [40]. There was scanning technique proposed, considered practical for novices to visualize the diaphragm. Apart from the need of examining the internal and external reliability of the technique and complex functional evaluation protocol development, the minimal expertise requirement and high degree of success suggest it might be potentially used by physiotherapists in daily practice, providing precision and reproducibility [41]. As medical universities use centers for medical simulation to improve qualifications and prepare for real-life situations, this settings could be potentially used to provide valid form of US training. Virtual-reality simulators could provide both training and standardized assessment of competence. Especially that they are commonly used in other specialties and demonstrate a potential for reproducible and objective assessment as well as effect on skill and behavior [42]. To ensure an appropriate use of diaphragm US, specific training for physiotherapists as well as examination protocols should be developed. They should be aimed not only at quantification of impaired muscle function, necessary as initial evaluation and follow-up assessment, but also at implementing ultrasound as a real-time support technique for respiratory muscle training, breathing exercise or chest physiotherapy.

One good example of possible application of diaphragm US in pulmonary physiotherapy could be IMT, requiring optimal resistance parameters adjustment. It appears beneficial especially in patients who cannot or do not want to adequately participate in standard spirometry test. The basis for determining IMT parameters is MIP, closely related to a measure of global respiratory muscle strength, and thus diaphragm strength. A relationship between diaphragm thickness and MIP gives potential to predict MIP based on US measurements. It allows for broader and more available diagnosing. Moreover, MIP has a potential to detect respiratory muscle weakness before forced vital capacity and total lung capacity do, therefore seems relevant also as a monitoring tool in the course of therapy [43]. It is also convenient to control the improvement of diaphragmatic motion with the use of US, in patients suffering from multiple rib fractures, unable to cough and breathe deeply as a result of severe pain sensation [10].

There is a progressive increase in the use of thoracic ultrasound by physiotherapists, indicating that this assessment tool is gaining in utility, probably thanks to its diagnostic precision and portability [44]. Still, there is limited research available regarding diaphragm US use in pulmonary physiotherapy interventions in COPD patients. It must be noted, that US should not replace typical physiotherapeutic assessment but rather provide added value to the range of diagnostic skills. Diaphragm ultrasonography may contribute to precise, reproducible practice, as accurate diagnosis of respiratory conditions is of importance when choosing the appropriate treatment, conducting therapy and monitoring its effectiveness [45].

## 5. Limitations

The limitations acknowledged regard language criteria. As the search was restricted to studies in English, the relevant studies in other languages could have been missed. Grey literature search was not conducted.

## 6. Conclusions

The review conducted suggests that excursion is the diaphragm US measure that is most often described in context of physiotherapy in COPD patients. The methodology applied, however, varies greatly making it difficult to compare results. In a presence of this finding, it appears, that there is an understandable need to develop standards of evaluation.

Basing on the studies identified, it is safe to conclude that diaphragm ultrasound may be the assessment method that effectively determines patient response to treatment, It is though worth considering, that US may not only be a measure of patient’s improvement, but also a tool facilitating clinical decision-making process. Future research studies could focus then on identifying which exact components of physiotherapeutic approach are essential for diaphragm functional improvement, as providing adequate level of evidence, regarding therapeutic treatment, should be of paramount importance as well.

## Figures and Tables

**Table 1:** Characteristics of the studies included in this review.

Author, Year	Study Design	Objective	Intervention	Participants	Techniques	Outcome Measure	Measure Details
Marques et al. 2019 [19]	Study protocol	To investigate the short-term effects of community based pulmonary rehabilitation programs	Pulmonary rehabilitation; 12-week	Recruited: 102	Comprehensive program	Thickness, cross sectional area, echointensity, and excursion	Not specified
Nair et al. 2019 [20]	Randomized crossover trial	To compare the effects of Diaphragmatic Stretch and Diaphragm Release techniques	Manual therapy; single-session	Recruited: 20 Completed: 20	Diaphragmatic Stretch Technique, Diaphragm Release Technique	Excursion	Sitting position B-Mode Both hemi-diaphragms Excursion: normal respiration
Crimi et al. 2018 [21]	Prospective observational study	To evaluate the changes in ultrasound measurements of diaphragmatic mobility and thickness	Exercise training; 12-week	Recruited: 37 Completed: 25	Endurance and strength training	Excursion; Thickness	Supine position M-Mode (excursion); B-Mode (kinetics) Right hemi-diaphragm Excursion: normal respiration; forced respiration; Thickness: normal respiration; forced respiration;
Corbelini et al. 2018 [22]	Prospective observational study	To verify diaphragmatic mobility improvement after in-patient pulmonary rehabilitation program and correlate the mobility loss to COPD severity	Pulmonary rehabilitation; 31 ± 8 days	Recruited: 46 Completed: 30	Comprehensive program	Excursion	Semi-recumbent position M-Mode (mobility) Right hemi-diaphragm Excursion: normal respiration; forced respiration;
Rocha et al. 2015 [23]	Randomized controlled trial	To verify if the Manual Diaphragm Release Technique improve diaphragmatic mobility after a single treatment, or multiple sessions	Manual therapy; 2-week	Recruited: 20 Completed: 19	Diaphragm Release Technique	Excursion	Semi-recumbent position M-Mode (mobility) Right hemi-diaphragm Excursion: forced respiration
Bhatt et al. 2012 [24]	Randomized crossover study	To determine the effects of volitional Pursed Lips Breathing Technique on exercise capacity	Breathing exercise; single-session	Recruited: 14 Completed: 14	Pursed Lips Breathing	Excursion	Supine position B-Mode (mobility) Right hemi-diaphragm Excursion: normal respiration; forced respiration;
Yamaguti et al. 2012 [25]	Randomized controlled trial	To investigate the effects of Diaphragmatic Breathing technique on thoraco-abdominal motion and functional capacity	Breathing exercise; 4-week	Recruited: 30 Completed: 30	Diaphragmatic Breathing	Excursion	Supine position B-Mode (mobility) Right hemi-diaphragm Excursion: forced respiration Cranio-caudal displacement of the left branch of the portal vein;

## Appendix A. Search Strategies

Medline Database

1. physiotherap*.ti,ab.
2. rehabilitat*.ti,ab.
3. “physical therapy”.ti,ab.
4. “manual therapy”.ti,ab.
5. “kinesio* taping”.ti,ab.
6. “exercise training”.ti,ab.
7. “respiratory muscle training”.ti,ab.
8. 1 or 2 or 3 or 4 or 5 or 6
9. diaphragm.ti,ab.
10. copd.ti,ab.
11. “chronic obstructive pulmonary disease”.ti,ab.
12. “obstructive lung disease”.ti,ab.
13. 10 or 11 or 12
14. ultrasonograph*.ti,ab.
15. ultrasound.ti,ab.
16. ultrason*.ti,ab.
17. 14 or 15 or 16
18. 8 and 9 and 13 and 17

Embase Database

1. physiotherap*.ti,ab.
2. rehabilitat*.ti,ab.
3. “physical therapy”.ti,ab.
4. “manual therapy”.ti,ab.
5. “kinesio* taping”.ti,ab.
6. “exercise training”.ti,ab.
7. “respiratory muscle training”.ti,ab.
8. 1 or 2 or 3 or 4 or 5 or 6
9. diaphragm.ti,ab.
10. copd.ti,ab.
11. “chronic obstructive pulmonary disease”.ti,ab.
12. “obstructive lung disease”.ti,ab.
13. 10 or 11 or 12
14. ultrasonograph*.ti,ab.
15. ultrasound.ti,ab.
16. ultrason*.ti,ab.
17. 14 or 15 or 16
18. 8 and 9 and 13 and 17

Pedro Database

Abstract & Title: Diaphragm
Therapy: respiratory therapy
When Searching: AND

Cochrane Database

#1. physiotherap* or rehabilitat* or physical therapy or manual therapy or kinesio* taping or exercise training or respiratory muscle training
#2. diaphragm
#3. copd or chronic obstructive pulmonary disease or obstructive lung disease
#4. ultrasonograph* or ultrasound or ultrason*
#5. #1 and #2 and #3 and #4
